# A Tet-On System for DRD1-Expressing Cells

**DOI:** 10.1371/journal.pone.0072681

**Published:** 2013-08-13

**Authors:** Fayi Yao, Paul D. Walker, Robert G. MacKenzie

**Affiliations:** 1 Department of Psychiatry and Behavioral Neurosciences, Wayne State University School of Medicine, Detroit, Michigan, United States of America; 2 Center for Molecular Medicine and Genetics, Wayne State University School of Medicine, Detroit, Michigan, United States of America; 3 Department of Anatomy and Cell Biology, Wayne State University School of Medicine, Detroit, Michigan, United States of America; Karolinska Inst, Sweden

## Abstract

Cells expressing the dopamine D1 receptor (DRD1) have significant functional roles in diverse physiological processes including locomotion and drug addiction. The present work presents a novel in vivo DRD1-Bacterial Artificial Chromosome (BAC) Tet-on system allowing for the inducible activation of tet-operated transgenes specifically within DRD1-expressing cells of transgenic mice. It is shown that the DRD1-rtTA BAC-driven expression of a tet-operated reporter is under tight regulation by doxycycline and is restricted to DRD1-expressing brain regions. The model will be a useful research tool in studies of movement and reward and associated pathologies such as Parkinson’s disease and addiction.

## Introduction

DRD1 is a seven transmembrane G protein-coupled receptor and one of two major dopamine (DA) receptor subtypes [1]. DRD1-expressing cells make up roughly half of the neuron population in the striatum [2]. Efferent projections from these cells form the direct striatal output pathway to the internal segment of the globus pallidus and the substantia nigra pars reticulata [3]. Other DRD1 cells are present in retina, olfactory tubercle, olfactory bulb, deep cortical layers, hippocampus, amygdala, hypothalamus and thalamus [4].

DRD1-expressing cells mediate a variety of important DA-modulated functions including locomotion and motivated behaviors. Hyperexcitability of DRD1 neurons is thought to underlie the dyskinetic response to L-DOPA treatment in animal models of Parkinson’s Disease [5,6]. Neurotransmission to and from DRD1-expressing cells, as well as chromatin remodeling within these cells, have been shown to control the reward and locomotor effects of cocaine and psychostimulants [7–10]. Research on these cells has been facilitated by the making of bacterial artificial chromosome (BAC) transgenic mice using modified *Drd1a*-BACs to express fluorescent reporters for identification of DRD1-expressing cells [11,12] and to express Cre recombinase for Cre/LoxP genetic manipulations specifically within DRD1 cells [13–15]. The present work describes a new mouse model that uses a modified *Drd1a*-BAC to drive an inducible Tet-On system for DRD1-expressing cells.

## Materials and Methods

### Ethics Statement

All animal procedures in this study were approved by the Wayne State University Institutional Animal Care Use Committee. Mice were maintained on 12-h light, 12-h dark cycles with normal chow and water *ad libitum*.

### Drd1-rtTA BAC transgenic mouse

**Figure 1:**
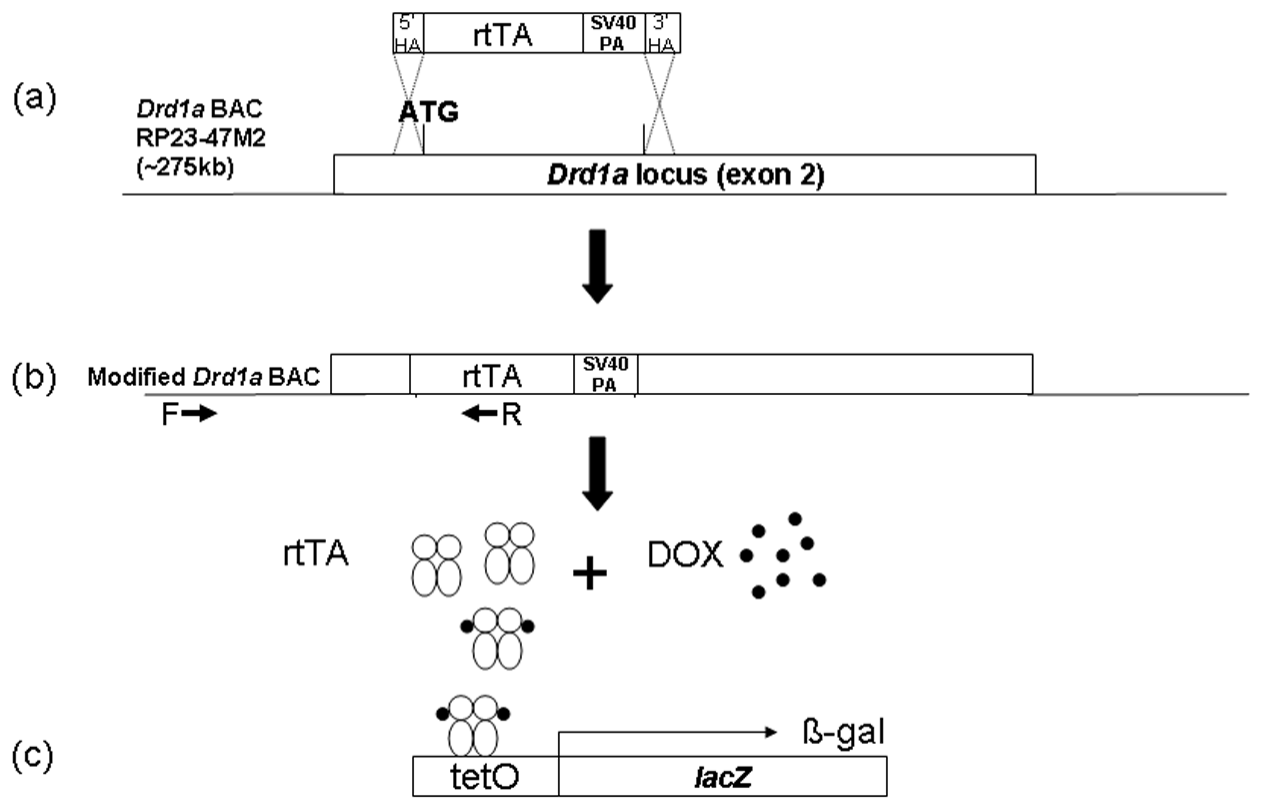
*Drd1a*-rtTA BAC TET-On system. (a) The *Drd1a* BAC (RP23-47M2) was modified by bacterial homologous recombination by insertion of the coding sequence for rtTA at the ATG start site for *Drd1a*. (b) The modified BAC makes rtTA in DRD1-expressing cells in transgenic mice. Arrows labeled F and R represent forward and reverse PCR primers for genotyping. (c) rtTA, in the presence of DOX, activates the tet operator to transcribe a tet-operated transgene.

A BAC designated RP23-47M2 was obtained from BACPAC resources of the Children’s Hospital Oakland Research Institute (CHORI). This BAC is approximately 274 kb and contains the loci for *Drd1a* and *Sfxn1*. The latter gene encodes a putative mitochondrial cation transporter protein, sideroflexin-1 [16]. Using the techniques of bacterial homologous recombination [17], a cassette containing the coding sequence for the reverse tetracycline-controlled transactivator (rtTA) was inserted at the ATG start site of the *Drd1a* locus (Figure 1a). rtTA is a fusion of the tetracycline repressor of the Tn*10* Tc resistance operon of *E. coli* and the C-terminal transactivation domain of VP16 from herpes simplex virus [18]. A plasmid construct was made containing 5’ and 3’ *Drd1a* homology arms (HA) of approximately of 280 and 386 bps, respectively, the cDNA of rtTA and the SV40 polyA signal sequence for homologous recombination into the *Drd1a*-BAC. The BAC was modified using the defective prophage system in which recombinogenic proteins were expressed from the modified DH10B *E. Coli* genome in a temperature-sensitive manner [19]. The plasmid was linearized and electroporated into the prophage-modified DH10B cells (strain EL250 from Neal Copeland) previously transformed by electroporation of the *Drd1a*-BAC. Recombination was promoted with a shift to the permissive temperature. The EL250 strain also supplied an arabinose-inducible Flp recombinase which was then used to remove the *Neo* selection marker. Successful BAC recombination and removal of *Neo* was verified by restriction analysis and sequencing. All constructs were validated by sequencing. All components made by PCR were sequenced and the modified BAC was validated by PCR and direct sequencing of the 5’ and 3’ insertion points. The purified BACs were run on a column, eluted with microinjection buffer and injected, uncut, into mouse zygotes of (C57BL/6 X SJL) F2 genetic background at the University of Michigan Transgenic Core to generate transgenic mice. Of 72 progeny, 9 founders were produced as determined by PCR genotyping of tail DNA using a primer pair specific for the 5’ *Drd1a*/rtTA junction: (F) 5’ – ATGCATGTATTTTAGGCTGTGTC -3’(R) 5’ – CGACTTGATGCTCTTGATCTTCC -3’

Transgenic mouse lines carrying the *Drd1a*-rtTA BAC transgene were crossed with reporter transgenic mice carrying tetO-*lacZ*. In bi-transgenic mice this approach (Tet-On) allows for activation of the tet operator by rtTA in the presence of doxycycline (DOX) [18] (Figure 1b and c). Therefore, during DOX administration, the *lacZ* gene produces β-galactosidase (β-gal) which can be detected through X-gal staining of the enzymatic product or immunostaining of the enzyme. DOX was simultaneously administered in both food (200mg/kg Bio-Serv, Inc., Frenchtown, NJ) and water (2mg/ml DOX in 1% sucrose). Bi-transgenic mice were also mated with *Drd1a*-(td) tomato mice (Jackson Laboratories stock #016204) to produce triple transgenic mice.

### X-gal staining

Mice were anesthetized using ketamine/xylazine and transcardially perfused with cold PBS followed by cold 4% paraformaldehyde (PFA) for 10 minutes. The brains were extracted, post-fixed in 4% PFA at 4^°^ C for 2 hours and immersed in 30% sucrose/PBS at 4^°^ C overnight. Brains were then embedded in OCT medium and stored at -80^°^ C. 50µm sections were cryocut and saved at -20^°^ C in anti-freeze medium. Sections were washed twice in washing buffer (PBS pH 7.4, 2 mM MgCl_2_, 0.01% sodium deoxycholate, 0.02% NP-40) for 10 minutes and incubated in X-gal staining solution (5mM potassium ferricyanide, 5mM potassium ferrocyanide, 1mg/ml X-gal, in washing buffer) at 37^°^ C overnight. Sections were washed in PBS 3 times for 5 minutes and dehydrated in alcohol in steps of 70%, 95% and 100% for 10 minutes each, followed by xylene for 10 minutes then mounted on slides with permount medium. Slides were viewed by bright field microscopy at 2.5X using a Leitz Laborlux 12 microscope (Wetzlar, Germany) equipped with SPOT Camera (SPOT Imaging Solutions, Sterling Heights, MI).

### Immunostaining

Mice were deeply anesthetized with sodium pentobarbital (210 mg/kg) and subjected to transcardial perfusion briefly with 0.1M sodium phosphate buffered saline (PBS, pH 7.4) followed by 50 ml 4% PFA in 0.1M PBS (pH 7.4). Dissected brains were immersed in fixative overnight prior to vibratome sectioning to obtain 50 µm coronal or sagittal sections. Free-floating sections were washed in 0.01M PBS (pH 7.4) followed by 1-hr incubation in blocking buffer containing 1% normal goat serum (MP Biochemicals, Aurora OH), 1% bovine serum albumin, 0.3% Triton X-100 in 0.01M PBS. Sections were incubated overnight in rabbit polyclonal antibody to β-gal (1:1000; 5Prime->3 Prime; Boulder CO) and mouse monoclonal antibody to tyrosine hydroxylase (TH) (1:200; gift of Dr. Gregory Kapatos, Wayne State University). Sections were washed in 0.01M PBS containing 0.02% Triton X-100(PBST) and incubated for 2-hr in blocking buffer containing goat anti-rabbit Dylight 488 and goat anti-mouse Dylight 549 secondary antibodies (each diluted 1:200; Jackson Immunochemicals, West Grove PA). Sections were washed in PBST, mounted onto glass slides out of 0.01M PBS, and coverslipped using Vectashield aqueous mounting media (Vector Laboratories, Burlingame CA). Other chemical sources not specified were Sigma-Aldrich (St. Louis, MO) and Fisher Scientific (Pittsburgh, PA). Sections were viewed by fluorescence microscopy using an Olympus IX-81 microscope equipped with a spinning disc confocal unit, using standard excitation and emission filters (Semrock) for visualizing FITC (Dylight 488) and rhodamine (Dylight 549). Images were captured using IPL imaging software (Silver Springs, MD).

## Results

**Figure 2:**
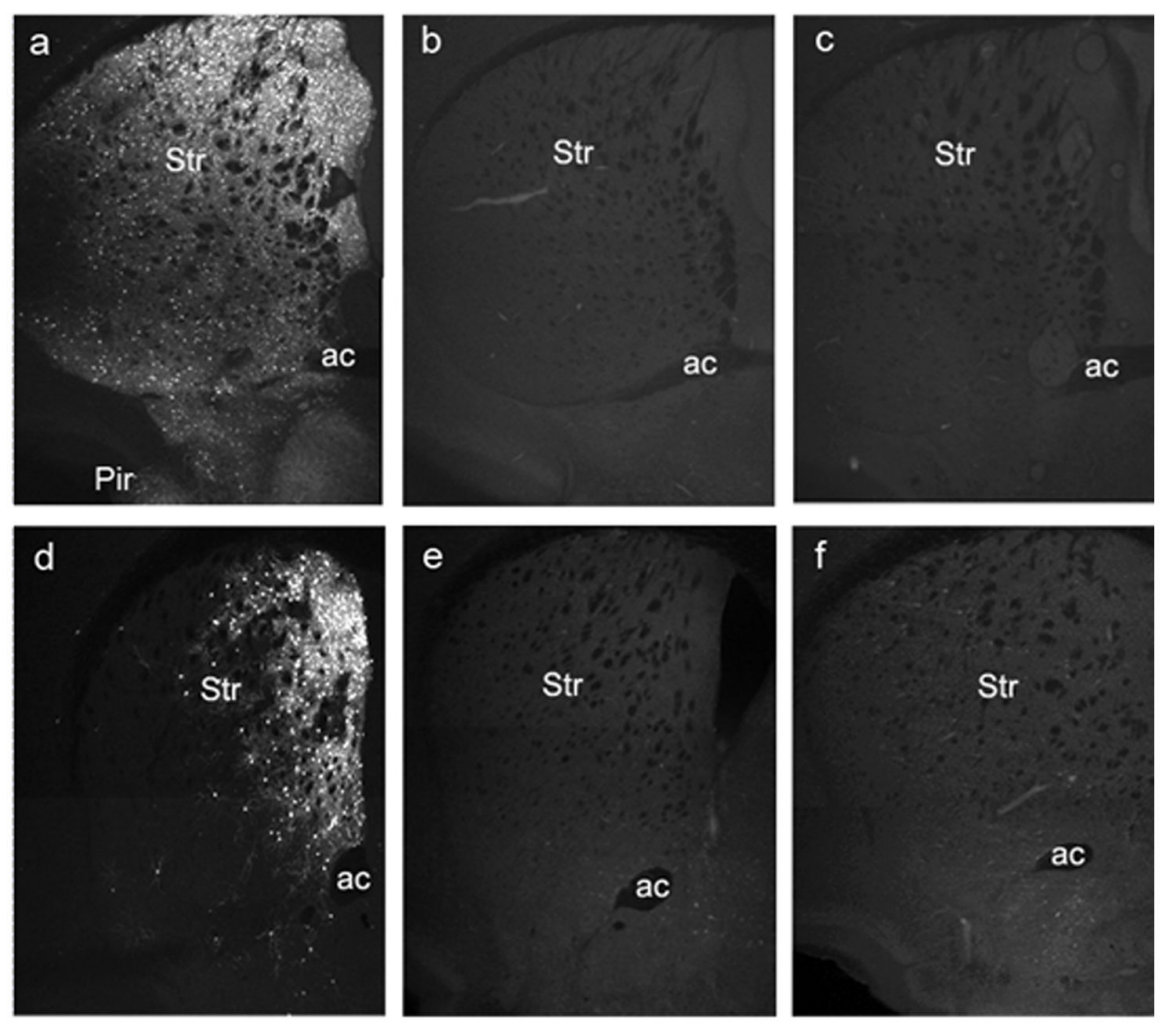
Expression of β-galactosidase is tightly regulated by DOX and requires the *Drd1a*-rtTA BAC. Coronal sections through the forebrain were immunostained for β-galactosidase. (a,d) *Drd1a*-rtTA BAC/tetO-*lacZ* bi-transgenic mice on DOX for 4 weeks, (b,e) *Drd1a*-rtTA BAC/tetO-*lacZ* bi-transgenic mice without DOX and (c,f) tetO-*lacZ* mice on DOX 4 weeks but without the *Drd1a*-rtTA BAC. Mice in a-c and d-f, respectively, were littermates. In a few bi-transgenic mice (d), β-galactosidase expression in striatum was primarily restricted to the dorso-medial region (see text). Abbr: Str, striatum; ac, anterior commissure; Pir, piriform cortex. Images were taken using a 4x objective.

One-month old littermate male mice were maintained on DOX or normal chow and water for 4 weeks, then perfused and tissue sections were analyzed by immunostaining for β-gal. Progeny from only one of the founders exhibited β-gal staining in the expected areas. All results in the present work come from this single founder. As shown in Figure 2a, immunostaining in bi-transgenic (*Drd1a*-rtTA BAC/tetO-*lacZ*) mice was restricted to regions known to express DRD1. Cell bodies throughout the striatum were labeled with a bias towards the dorsal-medial region of the structure. At 4x, the soma appear as bright dots and the above-background fluorescence through out the structure reflects the labeling of the dense network of processes projecting from the labeled cell bodies. The tight regulation by DOX in this Tet-On system was shown by the absence of any β-gal staining in bi-transgenic mice without DOX treatment (Figure 2b,e). The *Drd1a*-rtTA BAC transgene was required for DOX-induced expression of β-gal since no β-gal was detected in mice carrying only the tetO-*lacZ* transgene and exposed to DOX for 4 weeks (Figure 2c,f). In some bi-transgenic mice, β-gal expression in the striatum was primarily seen in the dorso-medial aspect of this structure (Figure 2d).

**Figure 3:**
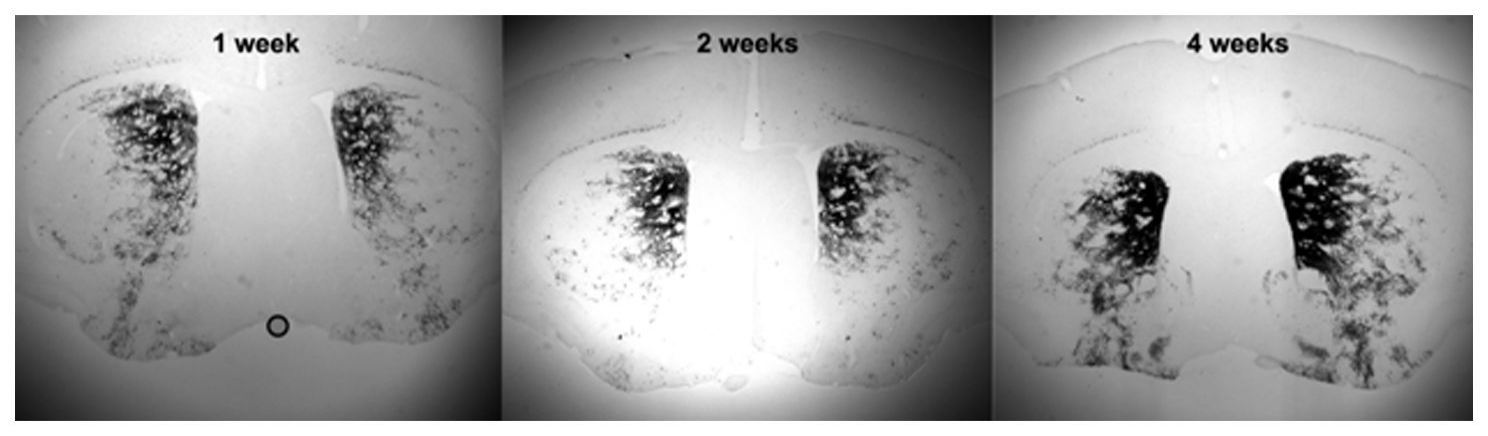
*Drd1a*-rtTA BAC/tetO-*lacZ* bi-transgenic mice express β-galactosidase after 1, 2 or 4 weeks of DOX treatment. Coronal sections through the forebrain show strong X-gal staining within 1 week of DOX treatment. X-gal staining in the striatum is most pronounced in the dorso-medial region of this structure. Images were taken using a 2.5X objective.

**Figure 4:**
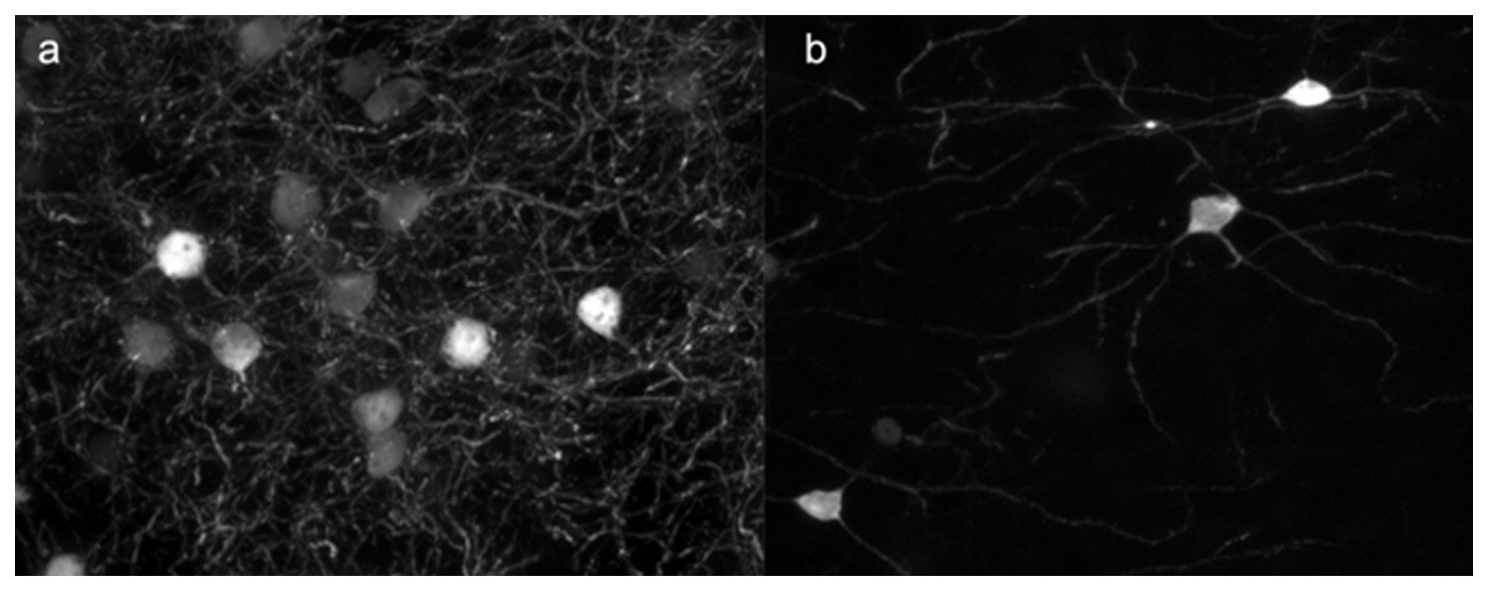
Expression of β-galactosidase is greatly reduced following a 2-week washout of DOX. Coronal sections of the dorso-medial region of striatum imaged at 40X following (a) 2-week DOX treatment or (b) 2-week DOX treatment followed by a 2-week period without DOX.

To determine the time required for DOX treatment to activate the Tet-On system, bi-transgenic mice were exposed to DOX for 1, 2 or 4 weeks while bi-transgenic littermate controls received normal food and water. As shown in Figure 3, DOX for 1, 2 or 4 weeks leads to rtTA-mediated activation of the tetO-*lacZ* transgene as demonstrated by X-gal staining. No controls exhibited any X-gal staining (not shown). Further, mice placed on DOX for 2 weeks but then removed from DOX for a 2-week washout period showed minimal expression of β-gal (Figure 4).

**Figure 5:**
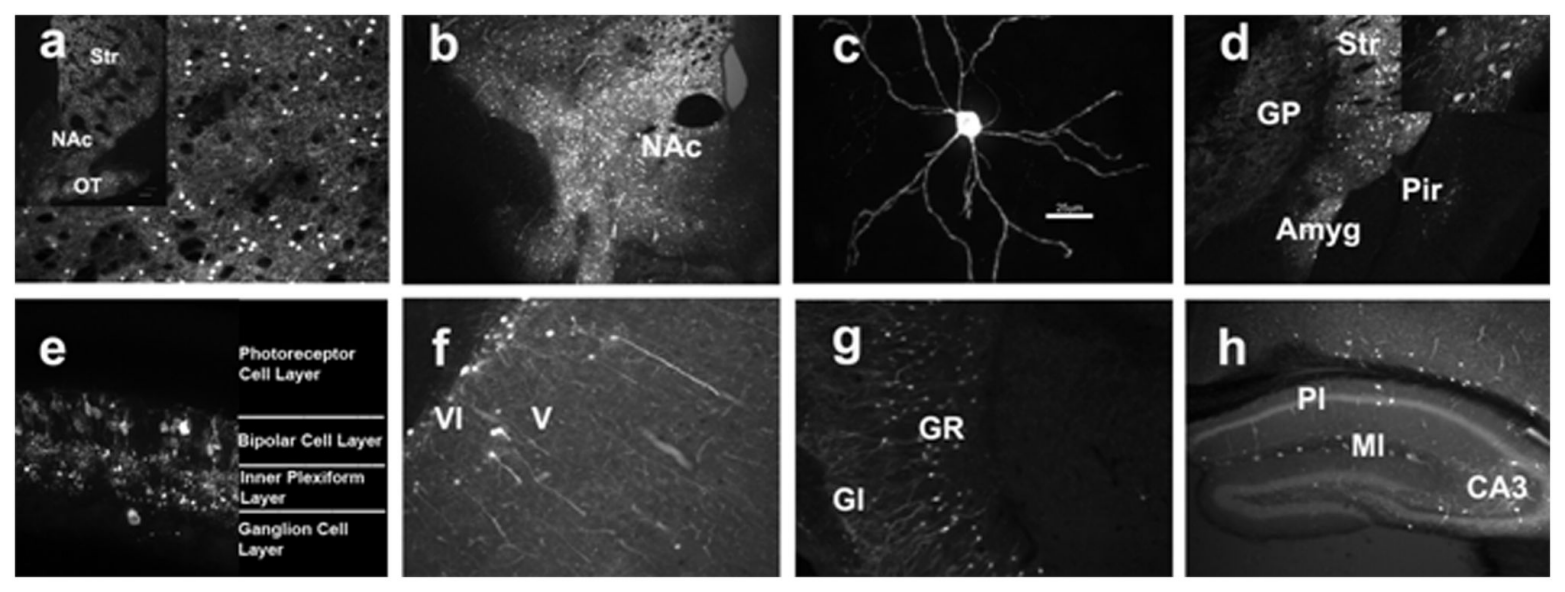
Immunostaining of β-galactosidase in bi-transgenic mice labels DRD1-expressing cells and neuronal processes in DRD1-expressing brain regions. (a) Coronal section of labeled cells in striatum at 10X. Inset is at 4X. (b) Intense labeling of cells in the core and shell regions of nucleus accumbens at 10X. (c) The arborization of a single medium spiny neuron in striatum at 40X. (d) Cells (4X) and cells showing processes (40X, inset) are labeled in amygdala. (e) Retinal bipolar cells with projections to inner plexiform layer and sporadic ganglion cells are labeled. (f) Expression in deep cortical layers V and VI (10X) and (g) in the granule cell layer of the olfactory bulb (10X). (h) Sporadic labeling of hippocampal cells in pyramidal, CA3 and molecular layers (4X). Abbr: Amyg, amygdala; CA3, CA3 field of the hippocampus; Gl, glomerular layer; GP, globus pallidus; GR, granule cell layer; Ml, molecular layer, NAc, nucleus accumbens; Pir, piriform cortex, Pl, pyramidal layer; Str, striatum.

In *Drd1a*-rtTA BAC/tetO-*lacZ* mice, the β-gal made in the DRD1-expressing neurons filled the neuronal processes and immunostaining for the enzyme resulted in a dense but highly defined network of dendritic processes in regions of high DRD1 expression such as dorsal and ventral (nucleus accumbens) striatum (Figure 5a and b). An example of the staining of both soma and processes in a single MSN is seen in Figure 5c taken from a lateral striatal site in a mouse in which β-gal expression was primarily restricted to dorso-medial striatum with sporadic labeling of cells in the lateral extension. Immunostaining was also strongly detected in the amygdala (Figure 5d); another known target of projections from midbrain DA neurons [20]. In addition, the reporter was detected in retinal bipolar cells and bipolar cell projections to the inner plexiform layer as well as sporadic retinal ganglion cells (Figure 5e), in deep cortical layers V and VI (Figure 5f), in the granule cell layer of the olfactory bulb (Figure 5g) and, sporadically, in the pyramidal, molecular and CA3 layers of hippocampus (Figure 5h).

**Figure 6:**
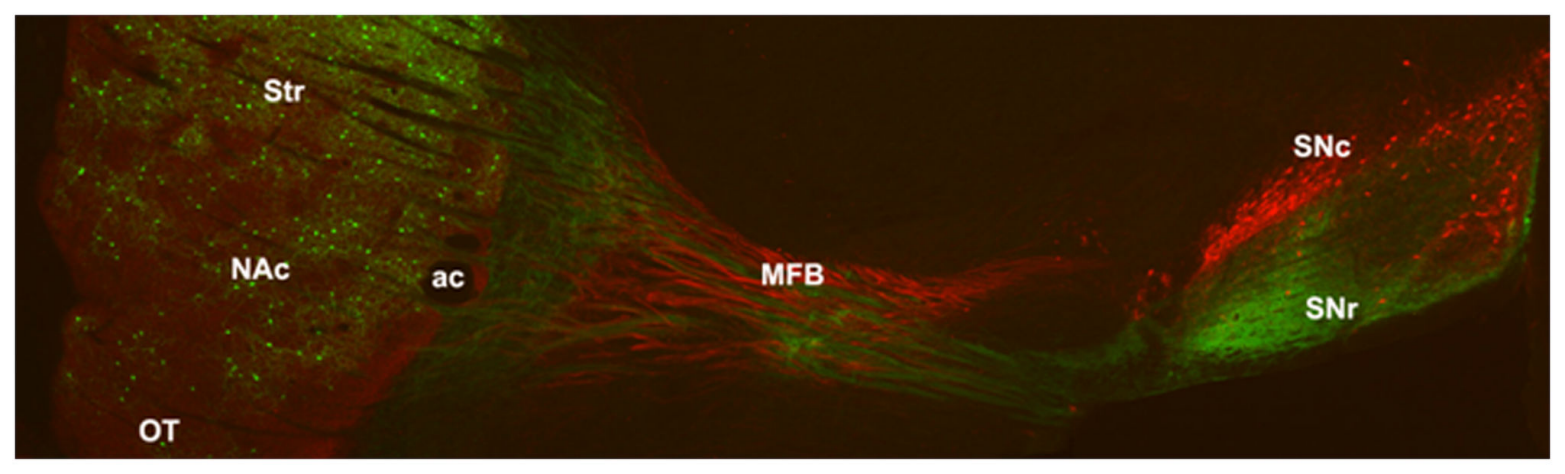
Nigrostriatal and striatonigral pathways. β-galactosidase immunostaining of sagittal sections from *Drd1a*-rtTA BAC/tetO-*lacZ* bi-transgenic mice on 4 weeks of DOX labeled the entire direct striatonigral pathway (green). The dopaminergic nigrostriatal pathway (red) was labeled by immunostaining for TH. Abbr: Str, striatum, NAc, nucleus accumbens, OT, olfactory tubercle, ac, anterior commissure, MFB, medial forebrain bundle, SNc, substantia nigra pars compacta, SNr, substantia nigra pars reticulata.

The β-gal labeling of processes of DRD1-expressing cells is evident in the elucidation of the direct striatonigral pathway from striatum, through the medial forebrain bundle to the substantia nigra, zona *reticulata* (Figure 6). The position of the direct striatonigral gabaergic pathway relative to the dopaminergic nigrostriatal pathway, originating from the substantia nigra pars compacta, is indicated by immunostaining for TH.

**Figure 7:**
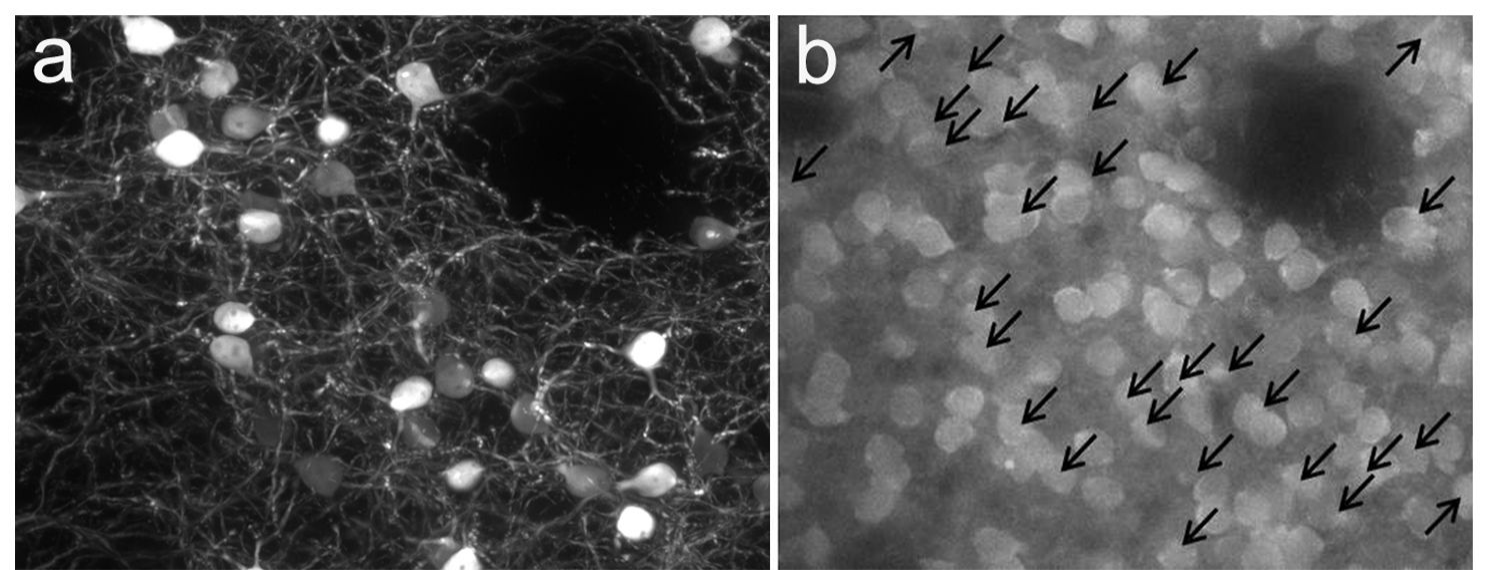
Co-localization of β-gal and (td) tomato expression in *Drd1a*-rtTA BAC/tetO-*lacZ/Drd1a*-(td) tomato triple transgenic mice. (a) Immunostaining of β-galactosidase in striatum of triple transgenic mice. (b) Direct red fluorescence of (td) tomato reporter in same section as (a). Arrows indicate (td) tomato labeled cells that express β-galactosidase. All β-galactosidase positive cells are also positive for (td) tomato.

To further validate that *Drd1a*-rtTA expression occurs only in DRD1-expressing cells, bi-transgenic *Drd1a*-rtTA BAC/tetO-*lacZ* mice were bred with *Drd1a*-(td) tomato mice which constitutively express the robust (td) tomato red fluorescent reporter from a modified RP23-47M2 *Drd1a* BAC transgene. The *Drd1a*-(td) tomato mice have been shown to label virtually all Drd1 MSNs [21]. Triple transgenic mice were exposed to DOX for 3 weeks and then euthanized, perfused, dissected, sectioned and immunostained for β-gal. Sections of striatal tissues at 20X and 40X from each of 3 different triple transgenic mice were examined by confocal microscopy. All β-gal positive cells within the 20X or 40X fields (a total of 296 cells) were positive for (td) tomato (Figure 7). However, only a subset of (td) tomato cells (19.5 ± 2.4%) were positive for β-gal.

## Discussion

The current report presents the first demonstration of an inducible Tet-On system for DRD1-expressing cells. The tetO-*lacZ* reporter shows that expression of rtTA from the modified *Drd1a*-BAC is restricted to cells within brain regions known to express DRD1 and is tightly regulated by DOX (Figures 2 and 4). Substantial expression of rtTA is achieved by 1 week treatment of DOX in food and water (Figure 3). Previously, an inducible system for DRD1-expressing cells was developed using the CreER approach [22]. In this case, a different BAC clone was modified (RP24-179E13) which does not contain the complete *Sfxn1* locus. In response to tamoxifen, expression of CreER in transgenic mice [15] appeared to be similar to that achieved for rtTA reported by β-gal in the DOX-treated *Drd1a*-rtTA mice. The similar DRD1-expression patterns and apparent health of the DRD1-expressing cells in *Drd1a-*rtTA mice indicate that the presence of the *Sfxn1* locus in the BAC clone had little impact which is supported by the eGFP and tomato reporter constructs that also used the *Sfxn1*-containing RP23-47M2 BAC clone [11,12]. The two inducible *Drd1a*-BACs represent complementary approaches to gene modification within DRD1-expressing cells with the Tet-On system capable of driving expression from tet-operated transgenes and the CreER system targeted to floxed alleles. The Tet-On system has the added advantage of reversibility as gene expression is turned off upon withdrawal from DOX.

Striatal expression of the *Drd1a*-rtTA BAC driven β-gal reporter does not appear to be as extensive as that demonstrated by enhanced green fluorescent protein (eGFP) and tomato reporters that are directly expressed from of the *Drd1a*-BAC [11,12] despite the use of the same BAC clone in all three systems. These differences might reflect different integration sites or copy number between the modified BACs or differences in the ability to assay or immunostain for β-gal on the one hand and detect directly expressed fluorescent products on the other. It is also possible that the promoter of tetO-*lacZ* undergoes silencing in a majority of cells when inactive during development as has been shown previously [23]. At worst, rtTA is made in a subset of DRD1-expressing cells.

Evidence for DRD1-specific expression of *Drd1a*-rtTA is as follows: First, the striatal cells labeled by β-gal are unequivocally MSNs by size and morphology. Second, the labeled cells are not DRD2-expressing cells since the indirect pathway originating from these cells to the globus pallidus externa (Gpe) is not labeled. The GPe would show increased fluorescence indicative of a terminal field if present as shown by others [12]. The only area that shows such increased fluorescence in the *Drd1a*-rtTA/tetO*lacZ* mice is the substantia nigra, pars reticulata, indicative of the direct pathway projecting from DRD1 MSNs and bypassing GPe as shown in Figure 5 and also by others using the *Drd1a*-(td) tomato reporter mouse [12,21]. Third, β-gal is detected in multiple brain regions and all these sites, without exception, are known regions of DRD1-expressing cells such as olfactory tubercle, nucleus accumbens, amygdala, hippocampus, the granule cell layer of olfactory bulb, deep layers of cortex, retinal bipolar cells and sporadic retinal ganglion cells. Finally, *Drd1a*-rtTA BAC/tetO-*lacZ*/*DRD1a*-(td) tomato show that all β-gal labeled cells in the striatum are also positive for the *Drd11a*-(td) tomato reporter which has been shown to specifically label DRD1-expressing cells [21].

The apparent limited activation of tetO-*lacZ* by *Drd1a*-rtTA to only 20% of DRD1-expressing MSNs might also limit the use of the *Drd1a*-rtTA mouse to direct expression of tet-operated transgenes to affect overall striatal function and/or behavior. If the limited β-gal expression is due to silencing of the tet promoter then different tet-operated transgenes integrated at different genomic sites might be less susceptible to silencing. Also, as others have shown, several approaches could be taken to decrease the silencing [23]. Moreover, even if rtTA-mediated transgene expression is limited to a minority population of direct striatonigral neurons, we are struck by the complex network of β-gal labeled processes from these cells that would likely have broad impact on striatal function given the high connectivity between MSNs and other cells [24,25]. Regardless of these considerations, it is clear that the *Drd1a*-rtTA mouse offers great utility for molecular, electrophysiological and anatomical approaches to evaluate the impact of post-developmental genetic manipulations on DRD1-expressing cells.

A recent study using retroviral monosynaptic tracing techniques has revealed a subset of striatal and nucleus accumbens cells, residing mostly in patches, which directly contact DA neurons in substantia nigra [26]. This result supports previous indications that patch neurons of the striatal patch-matrix organization can make direct contact with DA neurons [27]. Although, in the present study, the intense labeling of the substantia nigra by axons of the DRD1-expressing MSNs appears to be confined to the pars reticulata (as seen in Figure 6 and in other coronal sections not shown), scattered TH-positive neurons within *reticulata* are well-positioned to receive such direct contact and could account for the results of the tracing study. On the other hand, the tracing study did not profile the striatal cells that directly contact DA neurons so DRD1 expression of these cells has not been shown. Finally, in this regard, another study using optogenetic methods failed to provide evidence for direct connections from striatal to DA neurons in substantia nigra but used a variably expressed αCaMKII-tTA transgene to drive expression of the optogenetic components of the model [24,28]. In short, further work is required to establish the full nature of this direct striato-nigral DA pathway.

In summary, the present work presents a unique Tet-On system for DRD1-expressing cells. The inducible system is under tight regulation by DOX, allowing for genetic manipulation within DRD1-expressing cells in developing or adult mice and, consequently, the separation of potential developmental effects from those that impact on functionally mature processes. DRD1-expressing cells are involved in systems of reward and motor function and the present model, allowing for Tet-On manipulation of DRD1 cells within dorsal and ventral (nucleus accumbens) striatum will be useful in the study of these systems and their associated pathologies of Parkinson’s disease and drug addiction. *Drd1a-*rtTA mice have been deposited in Jackson Laboratories (stock # 018156) for general distribution.
